# Evaluating empathy level amongst the dental students using jefferson scale of physician empathy- health professional students

**DOI:** 10.1186/s12903-024-04267-w

**Published:** 2024-05-02

**Authors:** Beenish Fatima Alam, Raima Bashir, Talha Nayab, Talib Hussain, Bilal Zaman Babar, Syed Hassan Jan, Faisal Fahim

**Affiliations:** 1 Department of Oral Biology, Bahria University Dental College, Karachi, Pakistan; 2 Department of Dental Materials Science, Jinnah Sindh Medical University, Sindh Institue of Health Sciences, Karachi, Pakistan; 3 Department of Oral Biology, Women Dental College, Abbottabad, Pakistan; 4 Department of Dental materials, Women Dental College, Abbottabad, Pakistan; 5 Department of Oral Biology, HBS Medical and Dental college, Islamabad, Pakistan; 6 Department of General Education, Bahria University Medical and Dental College, Karachi, Pakistan

**Keywords:** Empathy, Health professionals, Dental students, Jefferson empathy scale, Pakistan

## Abstract

**Background:**

Empathy is described as one’s ability to perceive and apprehend another person’s feelings, situation, emotions, and problems as their own. Empathetic behavior increases patients’ satisfaction, reduces discomfort, and helps with patient’s satisfaction.

**Objective:**

To evaluate the psychometric properties of the Jefferson Empathy Scale and compare the measure of invariance within genders and amongst the public and private sector dental students.

**Method:**

This cross-sectional study utilized JSE-HPS version for research purpose. An exploratory factor analysis was performed to detect underlying factors. Reliability of the study tool was evaluated using Cronbach alpha test. Mann Whitney *U* test was used to compare the differences in scores between genders and among public and private university students while Student’s *t* analysis compared the scores according to different domains. The level of significance was ≤ 0.05.

**Results:**

Females demonstrated higher empathy levels (88.52 ± 14.19) along with private institute students (88.46 ± 13.98). Perspective taking and compassionate care domain was also scored highest by the females (31.73 ± 6.49 & 29.31 ± 6.22) and among second year students (33.30 ± 7.11 & 30.50 ± 7.16). PCA analysis extracted 4 factors namely (i) Health-care-provider’s sense of humor contributed to improved outcome (ii) Health-care provider’s understanding of patients’ feelings and of their families influences treatment outcomes (iii) Understanding body language is as important as verbal communication and (iv) Patients feel better when their feelings are understood, which accounted for the 59.51% of the total variance.

**Conclusion:**

The findings revealed that students from private institute and females demonstrated higher empathy score. Moreover, the Jefferson Scale of Empathy (JSE) was found to be a reliable and validated tool for assessment of empathy in our sample population.

## Introduction

Alfred Adler described empathy as the “Intellectual ability of someone to see with the eyes of others, hear with the ears of others and feel with the hearts of others”. The term empathy originated from two Greek words, “em” and “pathos,” signifying “feeling into” while its origin dates back to German language word “Einfühlung” which also translates as “Feeling into” [1]. Empathy is the ability to perceive and apprehend another person’s feelings, situation, emotions and problems as one’s own and is deemed to be a core aspect to strengthen any relationship [2]. With regards to patient care, empathy is believed to be a cognitive attribute and behavioral skill that enables one to understand patient’s pain and suffering and transfers these feelings to themselves with the intention to ease their discomfort and provide them relief [3]. 

Empathy has been considered as an important factor in delivering best health care facility to patients and achieving patients’ satisfaction and centeredness, which is one of the chief aims of 21st century health care system [4]. American Dental Education Association (ADEA) has also highlighted the significance of empathy, equivalent to interpersonal dental skills and has incorporated it into the list of Dental Clinical Competencies required to be achieved by every dental professional aspiring to practice in future [4]. Better treatment outcomes are believed to be linked with empathy, for practicing and learning health professionals [5]. Empathetic behavior towards patients increase their satisfaction, alleviates pain and discomfort, and improves compliance with physician’s instructions and recommendations. Thus, elevating the patient’s level of trust on physicians and health care systems and helps establish a good relationship between the two [5]. 

Empathy is considered an important competency for dentists. Supportive literature is available which reported that empathetic behavior of dentist was able to reduce patients’ fears and anxiety, enhanced patients’ trust, satisfaction and produced favorable treatment outcomes [6–9]. In short, Empathy helps improve basic communication skills of health care providers and builds a positive rapport between the two [10]. Dental students must practice empathy during their training years ensuring this vital attribute becomes an integral part of their personality.

A systematic review by Ridhi Narang summarized that, level of empathy declined among the dental students as they progressed in their academic years and had more patients’ exposure. Differences among male and female students regarding empathy were also observed [11]. The author proposed that educating students about empathy in behavioral sciences subject, can help retain their empathetic behavior over the years [11]. Differences in empathy scores between academic years were also revealed by Carilynne Yarascavitch et al. [8] and Priscilla Okhiabigie Ameh et al. [12] Improved and constantly rising empathy scores in higher academic years were transcribed by Torres-Martínez et al. in Chile [13], Muhammad Nazir in Saudi Arabia [14] and Katarzyna Mocny-Pachońska among polish students [15]. Higher empathy scores for senior year dental students as compared to juniors were also reported by Ghada H. Naguib et al. [16]

Previous studies conducted in Pakistan showed a significant decline in the mean empathy scores as the undergraduate students progressed in their academic years and between preclinical and clinical year students. (17–18) Sundas et al. found an increase in the empathy scores of undergraduate dental students after COVID-19 using Toronto Empathy Questionnaire (TEQ) underlining the fact, that fear of death was the contributing factor behind improved empathy scores [19]. 

Literature search elucidates that few studies had been conducted in Pakistan and worldwide which assessed and compared empathy scores among the dental students. Moreover, most of the research were majorly single centric and reported the responses from one institute only. The questionnaires employed to assess empathy scores were also different. Hence this study was undertaken to assess and compare the differences in the empathy scores of both public and private sector dental universities in Karachi using a validated and reliable tool designed especially to assess the levels of empathy among the Health care professional and Health care professional students known as Jefferson Scale of Physician Empathy (JSE). Secondly, we also aimed to evaluate the psychometric properties of the Jefferson Empathy Scale and compared the measure of invariance within genders and amongst the preclinical (first and second year) and clinical students (third and fourth) and trainees.

## Materials and methods

### Study setting and participants

This observational, cross-sectional study was conducted amongst the undergraduate dental students of private and public dental colleges in Pakistan. The research data was collected from July to November 2022. Undergraduate dental students from 1st to final years, and graduate students serving as dental residents (Post-graduate students) in different clinical departments were selected. Students from both private and public sector were involved as most of them belonged to different provinces, and had different cultural backgrounds.

In this study, students and residents were selected due to number of reasons: Firstly, the participants’ age were comparable with that of the students included in earlier studies, whose age varied from 19 to 23 years. Secondly, it was considered acceptable to compare responses of students who visited and interacted with the patients, as compared to those having no patient interaction. First and second year dental students despite having no clinical exposure were selected as pre-clinical group because they are being taught topics such as communication skills, behavioral sciences, child psychology, management of patient stress and anxiety in their curriculum. In second year, students have community trips, where they interact with school children and learn regarding both communication and examination skills before being exposed to patients. Third and final year students and residents formed our clinical group, as they are subjected to clinical exposure and deal with patients by applying prior learnt knowledge and experiences.

### Ethical approval

The Faculty Research Committee of the Institute approved the study whereas Institutional Ethical Review Committee granted the ethical approval for study, before the research commenced. The study adhered to the protocol listed in Declaration of Helsinki.

### Inclusion and exclusion criteria

Students from both genders, studying from 1st year to final years and dental residents, consenting and showing willingness to complete the questionnaire by participation were recruited in the study. Students with incomplete forms were excluded from the study.

### Study tool

A reliable and validated scale designed for a variety of Health care setting and comprising of psychometric properties of Empathy known as “The Jefferson Scale of Physician Empathy” (JSE) [20] was employed for data collection purpose. Dr. Mohammad Reza Hojat developed the JSE scale with an intend to evaluate the level of empathy amongst the health care professionals such as physician, healthcare students and paramedics involved in patient care in any clinical setting. The JSE scale has been used in 88 countries and translated into 54 languages due to its authentication and reliability in obtaining the desired empathy scores [21]. 

Three versions of the JSE-scale are available namely (i) “Medical students (S-version)”, (ii) “Health Professions (HP-version)” and (iii) “Health Professions students (HPS-version)”. The JSE - Health Professions students (HPS-version) was used in the present study [22]. 

This version was specifically designed for dentists, pharmacists, psychologists and nurses etc. It comprises of 20 questions which were scored using a 7 point Likert scale ranging from strongly agree through to strongly disagree. The score ranges from 20 to 140.

The self-reported questionnaire was divided into factors or domains. *Perspective taking* consists of question numbers 16, 13, 20, 15, 10, 02, 04, 09, 05 and 17, *Compassionate care* includes questions 11, 08, 07, 14 18, 01, 19, 12 and *Standing in the patient’s shoes* covers 2 questions: 3 and 6 respectively. Along with the questionnaire, demographic details regarding age, gender, academic year and institutes name were also undertaken.

### Procedure

This study was conducted in three phases. Firstly, the English version of questionnaire was evaluated for interpretation and understanding. Since English is the mode of teaching in our universities in Pakistan, hence adaptation of the questionnaire to our setting was not a concern, however all the items were thoroughly read and discussed with experts for any discrepancies in understanding. Secondly, validity of construct was performed, which comprised of the factor analysis and interrelation validity (association of scores with other domains) [23]. Last, reliability of the JSE-HPS was assessed to identify the internal consistency (precision of the instrument established on the standardization of all the items) and the reproducibility of the survey.

### Data collection

The data from private and public sector students and residents was collected on hardcopies by the researchers visiting the students during their free time. All the students from first year BDS to final years as well as residents were invited to participate in the study. The objectives of the study were explained and the questionnaires were distributed. All the participants gave verbal and signed written consent forms. The students not comfortable in filling the questionnaire were excluded. The questionnaire was self-reported and the students took 15–20 min to fill it. The questionnaires were collected and every student was thanked warmly for their contributions.

### Sample size

A sample size of Four hundred (400) was extracted using online OpenEpi software version 3.01 for cross-sectional studies. The confidence level was set at 95% with 5% margin of error. However, only 384 completely filled forms were included while incomplete forms where discarded from the study.

### Data analysis

SPSS version 23 (IBM Corp., Armonk, NY, USA) was used for result analysis. Principal component analysis (PCA) was performed using the varimax rotation method to calculate the correlations between the variables/factors of the JSE-HPS scale. Kaiser-Meyer-Olkin analysis assessed the overall index of the questionnaire. Bartlett’s Sphericity test evaluated the correlation among the factors/domains. The check of normality was conducted using Kolmogorov Smirnov test, following which factorial analysis was conducted. The Factor analysis revealed the eigen values. Descriptive statistics computed the mean and standard deviation (SD) values of JSE-HPE domains scores for different academic years for the students. Mann Whitney *U* test compared the mean empathy scores between genders and public and private sector university students while Student’s *t* test compared genders with the empathy domains. P-value < 0.05 was taken as significant.

### Reliability

Reliability of the study tool was assessed using the Cronbach’s alpha which determined the internal consistency of JSE, and the results were found to be acceptable (0.79). Thirty students were randomly selected to thoroughly fill the survey again after three weeks, to measure the test-retest reliability of the survey. The two assessments’ intraclass correlation coefficient was 0.82.

## Result

Table 1 displayed the mean and SD for PCA correlation and factor coefficient of JSE-HPS. To determine the relationships between the variables or factors of the JSE-HPS utilized, the principal component analysis (PCA) using the varimax rotation approach was employed. A Kaiser-Meyer-Olkin (KMO) analysis was performed to investigate the PCA criteria for factor structure identification. The data set was found to be appropriate for factor analysis because the KMO index for the current study was noted to be 0.86, which was more than 0.50.

The Factor analysis extracted four components with greater variance and eigenvalues out of the 20 JSE-HPS questions. These factors extracted by PCA, explained 59.51% of the total variance in this study.

**Table 1:** Factor coefficient, Mean (SD) for PCA correlation of JSE-HPS

	**Questions**	**Component**				**Mean (SD)**	r (Correlation)	
		1	2	3	4			
1.	Do you believe that empathy is important for effective communication with patients?	-0.603	0.451	0.157	0.035	2.15 ? 1.375	0.751	
2.	Do you believe that empathy can help lower the rates of patient litigation (Litigation is the process of settling a dispute in Court of Law?	-0.719	0.438	0.203	0.031	2.28 ? 1.387	0.758	
3.	Do you believe that having empathy can improve healthcare provider and patient relationship?	-0.699	0.476	0.204	-0.028	2.34 ? 1.425	0.755	
4.	Do you believe that empathy can lead to positive treatment outcomes?	-0.701	0.482	0.163	0.061	2.29 ? 1.374	0.684	
5.	Do you believe that empathy can improve the quality of patient dental care?	-0.672	0.446	0.180	0.017	2.37 ? 1.414	0.752	
6.	Do you believe that empathy is important for achieving patient satisfaction?	-0.662	0.530	0.149	0.100	2.17 ? 1.366	0.423	
7.	Health care providers understanding of their patients feelings and the feelings of their patients families do influence treatment outcomes	0.514	0.392	-0.058	-0.045	3.69 ? 1.638	0.751	
8.	Patients feel better when their health care provider understands their feelings	0.389	0.405	0.106	-0.651	3.81 ? 1.767	0.450	
9.	It is difficult for a health care provider to view things from patients’ perspectives	0.537	0.401	0.030	0.025	3.79 ? 1.65	0.452	
10.	Understanding body language is as important as verbal communication in health care provider - patient relationships	0.472	0.478	0.011	0.030	3.87 ? 1.723	0.540	
11.	A health care provider’s sense of humor contributes to a better clinical outcome	0.535	0.496	0.078	0.045	3.73 ? 1.642	0.516	
12.	.Because people are different it is difficult to see things from patients’ perspectives	0.570	0.196	0.189	0.341	3.84 ? 1.498	0.522	
13.	Attention to patients’ emotions is 2t important in patient interview (Complete History Taking)	0.487	0.474	-0.113	-0.218	3.67 ? 1.594	0.471	
14.	Attentiveness to patients’ personal experiences does it influence treatment outcomes.	0.441	0.524	-0.045	-0.001	3.63 ? 1.544	0.642	15.
15.	Health care providers should try to stand in their patients’ shoes when providing care to them.	0.611	0.408	0.013	0.318	3.68 ? 1.597	0.506	16.
16.	Patients value a health care provider’s understanding of their feelings which is Therapeutic in its own self.	0.569	0.355	0.174	0.161	3.81 ? 1.549	0.585	
17.	Patients’ illnesses can be cured only by targeted treatment; therefore health care providers’ emotional ties with their patients do 2t have a significant influence in the treatment outcomes	0.252	-0.169	0.644	-0.281	3.96 ? 1.66	0.566	
18.	Asking patients about what is happening in their personal lives is 2t helpful in understanding their physical complaints	0.229	-0.249	0.622	0.256	3.82 ? 1.536	0.590	
19.	Health care providers should try to understand what is going on in their patients’ mind by paying attention to their 2n-verbal ques and body language	0.105	-0.192	0.676	-0.291	3.73 ? 1.578	0.597	
20	I believe that emotion has 2 place in the treatment of medical illness	0.196	-0.313	0.651	0.193	3.58 ? 1.555		
% Variance		27.91	16.72	9.86	5.02			
Eigenvalue		5.582	3.34	1.97	1.00			

*Extraction Method*: Principal Component Analysis.

a. 4 components extracted

Factor 1 (a health care provider’s sense of humor contributes to a better clinical outcome) accounted for the largest share of the variance (27.9%), followed by Factor 2 (a health care provider’s understanding of their patients’ feelings and the feelings of their patients’ families do influence treatment outcomes), which reported a variance of 16.71%. Factor 3 (understanding body language is as important as verbal communication) accounted for 9.86% of the total variance, while Factor 4 (Patients feel better when their health care provider understands their feelings) had a variance of 5.02%. The correlation of JSE – HPS with the components was > 0.5, which depicted a good association.

Table 2 highlighted the differences in mean empathy scores amongst genders and students from different educational institutes. It was observed that higher empathy scores i.e. 88.52 ± 14.19 were identified for female participants as compared to the male participants 81.96 ± 12.12. Students from private university also demonstrated higher empathy levels 88.46 ± 13.98 in comparison with the empathy scores of public university students i.e. 81.20 ± 12.53 which was significantly different at a p-value of < 0.001**. (Table 2)

**Table 2:** Comparison of empathy score between gender and educational institutes

	Means of Empathy score	Std. Deviation	Std. Error Mean	U-value	P-value
Gender					
Female	88.5240	14.19438	0.83066	10144.00	< 0.001*
Male	81.9674	12.12250	1.26386		
**Educational Institution**					
Private sector	88.4671	13.98439	0.80206	8781.50	< 0.001*
Public sector	81.2000	12.53390	1.40133		

*Mann Whitney U test was applied

*P-value < 0.05 considered to be statistically significant

Findings from Table 3 revealed statistically significant difference with respect to domain scores between genders. The highest score was observed by females in the domain of perspective taking (31.73 ± 6.49) and compassionate care (29.31 ± 6.22) while the males displayed higher mean score in the domain of standing in patients shoes (5.64 ± 2.28). (Table 3)

**Table 3:** Comparison of empathy domain scores amongst gender

		N	Mean	Std. Deviation	95% Confidence Interval		t-value	P-value
					Lower Bound	Upper Bound		
Factor 1: Perspective Taking	Female	292	31.73	6.49	30.98	32.48	3.943	< 0.001**
	Male	92	28.77	5.54	27.63	29.92		
	Total	384	31.02	6.39	30.38	31.66		
Factor 2: Compassionate care	Female	292	29.31	6.22	28.60	30.03	5.226	< 0.001**
	Male	92	25.54	5.38	24.43	26.66		
	Total	384	28.41	6.23	27.78	29.03		
Factor 3: Standing in the patients shoes	Female	291	4.15	2.55	3.86	4.45	-4.993	< 0.001**
	Male	92	5.64	2.28	5.17	6.11		
	Total	383	4.51	2.57	4.25	4.77		

*Independant T test

Table 4 summarizes the multivariate analysis of domain scores of empathy with academic year of dental students. The standardized and unstandardized regression coefficient (β), along with 95% Confidence Interval (CI), and p-values of all the variables was computed. Year of academic education was considered to be a significant variable, whilst academic year an independent variable. Standing in patient’s shoes demonstrated a positive relationship with empathy. On the other hand, perspective taking and compassionate care showed negative correlation with empathy. (Table 4)

**Table 4:** Multivariate analysis of domain score with academic year of dental students

Model		Unstandardized Coefficients		Standardized Coefficients	t	p	95% Confidence Interval for B	
		B	Std. Error	Beta			Lower Bound	Upper Bound
	Factor 1: Perspective Taking	− 0.032	0.012	− 0.164	-2.664	0.008**	− 0.055	− 0.008
	Factor 2: Compassionate care	− 0.028	0.012	− 0.141	-2.273	0.024*	− 0.052	− 0.004
	Factor 3: Standing in patients shoes	0.051	0.024	0.106	2.118	0.035*	0.004	0.098

a. Dependent Variable: Year of Academic Education

*Multivariate regression analysis

## Discussion

The JSE had been used as a study tool to assess the level of empathy with respect to patient care and interaction. The JSE has received widespread acceptance on a global level since its first publication back in 2002 [24, 25]. The factor structure analysis by exploratory factor analysis (EFA) has been investigated in earlier research projects. To the best of author’s knowledge, this is the first research which has evaluated the four-factor model using the JSE-HPS amongst the Pakistani dental students. Numerous studies have studied the level of empathy amongst the university students in health professions, which includes medicine, nursing, pharmacy, or physiotherapy, using this scale [26–28]. 

The study tool’s reliability was measured using the cronbach’s alpha coefficient. In the current study, JSE-HPS tool demonstrated good internal consistency having α value of 0.79. Having an alpha value under 0.7 would have implied excessive heterogeneity whereas an alpha value above 0.9 would have denoted duplicate or redundant items. It should be noted that alpha value is basically an index reflecting the internal consistency which must be high to determine reliability, but it doesn’t provide data regarding the correlation of items using the factors [29]. Moreover, our internal consistency values were comparable with the prior studies using JSE-HPS [20, 30–32]. In addition, the stability of the outcomes and the internal consistency after the questionnaire was repeated, was found to be satisfactory, which was also in line with prior studies [33]. In the current study, we attained a score of 0.82, while Hojat et al. achieved reliability score of 0.65 after administering the study tool again after a period of 3–4 months [20]. 

In our study we analyzed the psychometric aspects of the study tool with the help of exploratory factor analyses (EFA) as performed in the prior studies [34]. Prior to conducting factorial analysis, normality check of the data was conducted using Kolmogorov Smirnov test and that test demonstrated significant values for all variables. Similarly, investigation by Diaz et al., also detected the normality of the data using Kolmogorov Smirnov test [35]. Once the normality of data was checked, further analysis using EFA was performed. Analysis using the EFA revealed a four-factor structure in our research. Comparably, the research conducted by Hojat et al. and Preusche et al. both noted four-factor models [20, 36]. The research conducted by Tavakol et al. and Alcorta-Garza et al. indicated a three-factor model, which differs from our findings [34, 36]. These discrepancies may be explained by the fact that the majority of researchers that observed three-factor models had additionally subdivided their items into domains that included perspective taking, compassionate care, and standing in the patient’s shoes [34, 37]. While in our study although we had observed four factor models, only one item (Patients feel better when their health care provider understands their feelings) was included in this fourth factor. This factor could be considered as “negative/no influence of moderating factors and (empathic) techniques/skills on process/outcome”, as reported previously by Preusche et al., despite identifying four items associated with the fourth factor [36]. Furthermore these differences between studies could also be because of cultural differences that exists between countries, religious beliefs, and how the people had perceived the items.

In this study “health care provider’s sense of humor contributes to a better clinical outcome” yielded factor loading (*r* = 0. 0.516) and low communality (h2 = 0.43) values. This item’s communality value was relatively low, indicating that humor, which has been used for patient care in Western countries, had a positive impact. Because of this, it was considered essential to stress on the importance of humor while interacting with patients [38]. In clinical settings, humor helps ease stress and reduces fear, which enhanced empathetic engagement between patient and physician [39]. The next item, “Health care providers’ understanding of their patients’ feelings and the feelings of their patients’ families do influence treatment outcomes,” revealed a low communal value. This could be further improved if treating physicians realize that empathizing with patients was a prerequisite for providing effective care, which suggested that having compassion for patients should be viewed as a basic component of their competency [40]. Third factor which also resulted in lower communal value was “understanding body language is as important as verbal communication”. Given the fact that nonverbal communication is expressed through the body’s movements, and the physicians must recognize the significance of body language. This suggested that several nonverbal clues denoted power, subordination, openness, fear, discomfort, and nervousness [41]. Last item “Patients feel better when their health care provider understands their feelings”, revealed (h2 = 0.75) Even though our results for this item are somewhat better, but physicians can offer better treatment outcomes if they understand how important it is to comprehend their patients’ feelings.

In our study, female students were greater in number than males. According to our findings, women performed noticeably better than men and had an overall higher score, as identified by several studies and cultural contexts [28, 31]. Perspective-taking, which has been viewed as the cognitive component of empathy, was measured as the first dimension, while compassionate care as the second dimension. Women performed better in both of these domains. Similar results, indicating a higher score in women, have been noted in numerous investigations conducted in different countries using different versions [42–44]. This could be attributed to the influence of education and culture, which has already been extensively discussed in prior studies. (45–46)

Year wise comparison of mean empathy scores in different domains yielded differences which supported the findings from previous studies [24, 47, 48]. The present study demonstrated a positive relationship between higher levels of empathy and employment within patient-care-related sectors. Differences with respect to emotional intelligence or personality could explain the disparities in the student performance identified studying in different years. These disparities may be attributed to a variety of factors, including economic status, cultural beliefs, family backgrounds, being exposed to the real world as one ages, and encountering different situations [11, 14, 37]. Furthermore, work-related burnout resulting from exposure to a stressful and unfavorable working environment could also influence doctors’ empathic behavior toward their patients. [49–50]

Prior research showed that feelings of empathy tend to decline during the training of health care professionals, including those in medicine and dentistry. Majority of students are usually elated and enthusiastic when they first start their medical studies; however, it’s levels declines over the course of time [51]. Similarly, in the current study during the first and second year, empathy scores in the three domains were higher while the mean scores for final year students and residents, declined with increased patient’s interaction. These outcomes are in line with prior studies where authors identified, that third year of study marks as a turning point for students’ empathic behaviors, as during this period they enter their clinical training phase and a decline in students’ empathy levels starts to occur [52]. 

### Limitations

The data of the study was limited with respect to sample size as it incorporated students from a single private and public sector institute and further research involving multi-centers and larger sample sizes are required for generalization of results. 1st and 2nd year students despite having no clinical interaction with patients formed part of the study sample because they had been taught empathy during their academic years. The differences observed in the study could thus be utilized to address these issues in future. The scores depicted by the students may present recall biasness which can be eliminated by employing more comparable scales such as Toronto Empathy questionnaire (TEQ) and Jefferson Scale of Physician Empathy (students’ version; JSPE-S) to validate and strengthen the findings, instead of a single scale incorporated in the study to measure empathy. The cross-sectional nature reflects only perceptions of the respondents and does not provide any causal association with the responses.

## Conclusion

This study identified higher level of empathetic behavior of students enrolled in the private dental institute. Additionally, females demonstrated higher empathic thinking and compassionate care behavior towards patients whereas male students demonstrated significantly higher scores in “Standing in the patients shoes” domain. It is thus imperative to foster the empathetic behavior in male students as they represent a large population of practicing practitioners. Modifications in teaching approach which includes early non- clinical interaction of dental students (during their 1st and 2nd years) with patients limiting them to observing history taking, demonstrations and oral exam adhering to principles of professional ethics, would help dental students develop empathic skills whereby, making them understand the behavior of patients and help them in gaining patients’ trust for better treatment outcomes.

## Data Availability

The data from the current study are available from the corresponding author on reasonable request.
